# Supplementary material for: Associations of Neighborhood Walkability with Sedentary Time in Nigerian Older Adults
Source: Int J Environ Res Public Health. 2019 May 28;16(11):1879. doi: 10.3390/ijerph16111879 (PMC6603618; doi:10.3390/ijerph16111879)
Supplement: Supplementary file 1 [file ijerph-16-01879-s001.pdf]

Table S1. Associations between perceived neighbourhood environment attributes with sedentary time (min/day) on a weekday and a weekend day

Environmental Attributes	Effect	Weekday-Environmental-Attributes Models			Weekend-Environmental Attributes Models		
		B	95% CI	*p*-Value	B	95% CI	*p*-Value
Residential density	Main	−0.285	−0.584, 0.013	0.061	−0.204	−0.487, 0.080	0.159
	Women-specific	-	-	-	-	-	-
	Men-specific	-	-	-	-	-	-
Proximity to destinations	Main	−116.558	−159.694, −74.421	<0.001 **	−56.478	−99.889, −13.070	0.011 *
	Women-specific	−109.231	−179.069, −39.394	0.003 *	-	-	-
	Men-specific	−103.314	−158.053, −48.574	<0.001 **	-	-	-
Access to services and places	Main	−85.679	−134.507, −36.851	0.001 *	−25.154	−73.987, −21.679	0.282
	Women-specific	-	-	-	-	-	-
	Men-specific	-	-	-	-	-	-
Street connectivity	Main	−10.687	−49.480, 28.106	0.587	−3.847	−33.484, 41.178	0.839
	Women-specific	−6.092	−54.901, −42.717	0.804	-	-	-
	Men-specific	46.555	2.575, 90.536	0.038 *	-	-	-
Walking infrastructure and safety	Main	−35.514	−78.507, 10.200	0.587	−26.559	−69.142, 16.023	0.220
	Women-specific	−3.505	−54.500, 47.490	0.892	-	-	-
	Men-specific	−39.178	−77.598, −0.758	0.046 *	-	-	-
Aesthetics	Main	−11.103	−50.000, 27.793	0.574	−11.656	−49.027, 25.715	0.539
	Women-specific	-	-	-	-	-	-
	Men-specific	-	-	-	-	-	-
Traffic safety	Main	−89.813	−131.063, −48.571	0.001 *	−74.184	113.693, −34.601	<0.001 **
	Women-specific	−51.597	−98.427, −4.766	0.031 *	−59.298	−106.641, −11.955	0.015 *
	Men-specific	−79.234	−126.418, −32.050	0.001 *	−62.469	−106.189, −18.749	0.006 *
Safety from crime	Main	−50.339	−78.826, −21.853	0.001 *	−25.684	−53.398, 0.029	0.048 *
	Women-specific	−28.280	−58.665, 2.105	0.068	-	-	-
	Men-specific	−20.295	−46.856, 2.625	0.132	-	-	-
Overall walkability index	Main	−10.981	−16.897, −5.065	<0.001 **	−7.836	−13.540, −2.132	0.007 *
	Women-specific	-	-	-	-	-	-
	Men-specific	-	-	-	-	-	-

B = regression coefficient; 95% CI = 95% confidence intervals; - =Not applicable because no significant moderating effect of sex was found. For environmental attributes with significant sex moderating effects, sex-specific associations (men- and women-specific) are reported. All regression coefficients were adjusted for participants' age, sex, marital status, education, employment and neighbourhood types.
